# miR-128 Is Implicated in Stress Responses by Targeting MAFG in Skeletal Muscle Cells

**DOI:** 10.1155/2017/9308310

**Published:** 2017-09-12

**Authors:** Rocco Caggiano, Fabio Cattaneo, Ornella Moltedo, Giovanni Esposito, Cinzia Perrino, Bruno Trimarco, Rosario Ammendola, Raffaella Faraonio

**Affiliations:** ^1^Dipartimento di Medicina Molecolare e Biotecnologie Mediche, Università di Napoli Federico II, Napoli, Italy; ^2^Dipartimento di Scienze Biomediche Avanzate, Università di Napoli Federico II, Napoli, Italy; ^3^Dipartimento di Farmacia, Università degli Studi di Salerno, Fisciano, Salerno, Italy; ^4^CEINGE-Biotecnologie Avanzate s.c. a r.l, Napoli, Italy

## Abstract

MAFG (v-Maf avian musculoaponeurotic fibrosarcoma oncogene homolog G) is a bZIP-type transcriptional regulator that belongs to the small MAF (sMAFs) protein family. By interacting with other bZIP transcription factors, sMAFs can form homo- and heterodimers governing either repressive or activating transcriptional functions. As heterodimeric partner of Nrf2, MAFG positively influences the ARE-dependent antioxidant/xenobiotic pathways, at least in condition of a correct MAFG:Nrf2 balance. MicroRNAs (miRs) participate to different regulatory networks being involved as fine-tuning regulators of gene expression. However, the connections between cellular surveillance to stresses mediated by MAFG:Nrf2 and miR regulations are not well understood. Here, we explored the impact of miR-128 in expression of genes related to stress response. Bioinformatic predictions coupled with functional analysis revealed the presence of miR-128 binding site in the 3′UTR of MAFG. Ectopic miR-128 expression correlated with reduced expression of endogenous MAFG-dependent genes and negatively affected ARE-mediated molecular phenotype based on Nrf2 activity. Indeed, miR-128 impairs redox-dependent pathways induced in response to oxidative stress. Moreover, in condition of hypoxia, MAFG induction correlated with reduced levels of miR-128. This lead to increased mRNA levels of HMOX-1 and x-CT for blunting stress. Overall, these findings identify MAFG as novel direct target of miR-128.

## 1. Introduction

In response to oxidative and xenobiotic stresses, cells activate numerous defense systems associated with both enzymatic and not enzymatic activities. The events underlying these redox-related responses are accomplished by a tight regulation of gene expression patterns involving multilayered regulatory mechanisms [1, 2]. Two transcription factors shown to be highly involved in the regulation of numerous antioxidant and detoxifying genes at transcriptional levels are Nrf2 [nuclear factor (erythroid-derived 2)-like 2] [3–5] and MAFG (v-Maf avian musculoaponeurotic fibrosarcoma oncogene homolog G) [6]. In particular, in the form of heterodimer, the complex Nrf2:MAFG binds to and activates the transcription of antioxidant/xenobiotic genes harboring antioxidant responsive elements (ARE)/electrophile responsive elements (core ARE: TGACNNNGC), located in their transcription regulatory sequences [7, 8].

The proteins of the MAF family, described for the first time as a viral oncogene in their prototype v-Maf, are transcription factors, which are widely known to participate in gene expression regulation [9]. The MAF family consists of 7 members, which are grouped into “large” (c-Maf, MafA, MafB, and Nrl) and “small” (MafG, MafK, and MafF) Maf subfamily, based on their size [6]. Each member of the MAF family harbors a basic-leucine zipper (b-ZIP) domain involved in DNA binding and in dimer formation, either with themselves or with different b-ZIP transcription factors, in particular Nrf2 [10, 11]. In addition to the b-ZIP domain, large Maf proteins also possess an acidic transcriptional activation domain (TAD) that, on the contrary, is absent in the small Mafs (sMafs). For this reason, the regulatory activity of small Mafs on gene transcription can be positive or negative, depending on their specific partner and on the promoter context. In general, homodimers of sMafs lacking TAD (i.e., MafG:MafK) repress gene transcription by binding to the Maf recognition element (MARE: TGCTGACTCAGCA) [6, 12]. The heterodimers with cap ‘n' collar (CNC) proteins such as p45 NF-E2 [13] or with NF-E2-related factors (Nrf1, Nrf2, and Nrf3) [14, 15] as well as with the Bach (BTB and CNC homology) factors (Bach1 and Bach2) [16], unlike homodimers, can either function as transcriptional activators or repressors [6]. Among these, as mentioned before, the Nrf2:sMaf heterodimers promote transcription and represent the most relevant means for adjusting a multitude of intracellular protein levels in response to oxidative/electrophilic stresses [5]. Under basal unstressed conditions, Nrf2 is polyubiquitinated and targeted to 26S proteasome by the Kelch-like ECH-associated protein 1(Keap1)-Cul3 E3 ligase complex [17]. Stresses provoke dissociation of Keap1 through modification of specific cysteine residues [18]. Thus, Nrf2 can migrate into the nucleus, where in association with sMaf, in particular MAFG, binds to the promoters of target genes harboring ARE sequences to activate their transcription [8].

sMafs also interact with HIF-1*α* to positively regulate hypoxic responses [19]. On the other hand, Bach1:sMaf heterodimers act as negative regulator of the HMOX-1 gene and its role is supported by genetic data [20]. In addition, Fang and colleagues recently demonstrated that high MAFG levels driven by BRAF (V600) recruit Bach1 as partner along with CHD8, a chromatin remodeling factor, and DNMT3B, a DNA methyltransferase, to trigger epigenetic silencing of genes frequently hypermethylated in melanoma and colorectal cancer [21].

In the last decades, microRNAs (miRs) have added a new layer of complexity to the regulatory mechanisms underlying gene expression [22, 23]. miRs are small endogenous noncoding RNAs that downmodulate protein expression at posttranscriptional level. By interacting with specific regions, generally localized in the 3′-untranslated regions (UTRs) of transcripts, miRs mediate repression of mRNA translation or stability [24, 25]. Given that each single miR could target multiple transcripts and often a miR regulates several targets of the same pathway, they supervise cellular signaling and networks involved in fundamental biological processes, including stress responses [26–28].

Numerous studies have shown that miR-128 is involved in different cellular processes such as differentiation, apoptosis, senescence, and metabolism [29–32]. Here, we investigated its role in the stress/antioxidant networks regulated by the heterodimeric complex Nrf2:MAFG. We demonstrated that miR-128 by targeting MAFG influences stress responses mediated by ARE-dependent genes.

## 2. Materials and Methods

### 2.1. Cell Cultures and Reagents

Human embryonic kidney HEK293 cells and mouse myogenic C2C12 cells were obtained from American Type Culture Collection (ATCC, Manassas, VA, USA). Cells were cultured in Dulbecco's modified Eagle's medium (DMEM) (Thermo Fisher SCIENTIFIC, Italia, Monza, Italy) supplemented with fetal bovine serum (FBS) (Invitrogen) at 10% for HEK293 and 20% for C2C12 cells. To induce differentiation of C2C12 cells, culture medium was replaced to near-confluent cultures (about 90%) with DMEM containing 2% horse serum (Thermo Fisher SCIENTIFIC).

Hypoxia (2% O_2_) for 4 h was induced when C2C12 cells were at 85% confluence. After being washed three times with phosphate-buffered saline (PBS), cultures were transferred to a 37°C incubator within a hypoxic chamber (93% N_2_, 5% CO_2_, 2% O_2_), and culture medium was replaced with a saline buffer that was prebubbled for five minutes with the same gas mix, to provide an average O_2_ pressure (14.7 mmHg) equivalent to that in the ambient air of the chamber.

Treatments with diethylmaleate (DEM) (Sigma-Aldrich, Milan, Italy) were performed for 2 h (HEK293) or 4 h (C2C12) using a final concentration of 200 *μ*M in complete culture medium.

### 2.2. Plasmid Constructs and Transfections

The plasmid expressing miR-128 (pCMV-miR-128) was obtained by cloning the human ARPP21 intronic region of 284 bp encompassing the pre-miR-128-2 sequence with 100 bp upstream and downstream flanking sequences into pRcCMVneo vector. This fragment was prepared from human IMR90 DNA by PCR using the following oligonucleotides: 5′-ACgTAAAAgCTTAAgAAggCTATTgACAATCCAg-3′ and 5′-CgACATATCTAgATTTggTCAgCAggAATgaCAC harboring HindIII and XbaI restriction sites, respectively. The identity of the cloned region was established by digestion and confirmed by sequencing. The expression of mature miR-128 was evaluated by Northern blot (Supplementary Materials available online at https://doi.org/10.1155/2017/9308310) in HEK293 cells and miR-128 levels measured by reverse transcription-quantitative real-time PCR (RT-qPCR).

The luciferase constructs were produced by cloning the 3′UTR regions with the miR-128 site/s of candidate genes into the pGL3-control vector, downstream the Firefly luciferase gene (Promega, Madison, WI, USA). The 3′UTR fragment of MAFG was amplified by PCR using human IMR90 DNA with primer pairs containing XbaI sites: 5′-ATCATTCTAGAGGATCCATGCAGGCATGCTGGCTCC-3′ and 5′-ATCATTCTAGATTCAAGCTACTATCAGACAATGT-3′. The 3′UTR of Nrf2 was obtained from the full length cDNA (I.M.A.G.E.: 4548874) by using the following primers with Xba sites: 5′-ACGTACTCAGATTTAGGAGGATTTGACCT-3′ and 5′-ACGTACTCAGATAACAGTCATAATAATCCTTTATTA-3′. The orientation of the cloned fragments was established by digestion and confirmed by sequencing. The pGL3 constructs with the reverse orientation were used as negative controls. All the plasmids bearing the 3′UTR fragments were transfected in HEK293 cells with Lipofectamine 2000 Reagent (Thermo Fisher SCIENTIFIC) in 24-well plates (40.000 cells/well), according to the manufacturer's instructions, and cotransfected with synthetic pre-miR-128 (ID: PM1176) or pre-miR-negative control number 1 (100 nM) (Thermo Fisher SCIENTIFIC) as well as with pCMV-miR-128 or pCMVneo (ratio 1 : 5). Transfections of cells with synthetic pre-miR-128 or pre-miR negative control were performed as previously described [33].

The plasmid expressing the FLAG-tagged Nrf2 and the plasmids GSTA1-LUC, x-CT-LUC, and NQO1-LUC bearing the promoters of the respective genes driving the expression of luciferase reporter gene have been described previously [34, 35].

### 2.3. Luciferase Reporter Assay

The pGL3-3′UTR constructs (100 ng) were cotransfected with the Renilla luciferase reporter plasmid (20 ng) as an internal control using Lipofectamine 2000 (Thermo Fisher SCIENTIFIC) in HEK293 cells in the presence of miR-128 or negative control. Luciferase activity was measured at 24 h after transfection using a dual luciferase reporter assay (Promega) according to the manufacturer's instructions and performed on a 20/20^n^ Luminometer (Turner BioSystems, Sunnyvale, CA, USA). Relative luciferase activity was calculated by normalizing the Firefly luminescence to the Renilla luminescence and then calculated relative to the control. C2C12 cells plated in 24-well plates (30.000 cells/well) were cotransfected with the GSTA1-LUC, x-CT-LUC, and NQO1-LUC constructs (500 ng) and FLAG-Nrf2 or empty vector (60 ng) and simultaneously with miR-128 expressing plasmid or empty vector (500 ng) using Lipofectamine® LTX Reagent (Thermo Fisher SCIENTIFIC) according to the manufacturer's instructions. All the transfections also contained a Renilla luciferase construct (40 ng) for internal normalization. Cells were then harvested at 36 h after transfections, and luciferase activity was determined as described above.

### 2.4. Western Blotting

Total proteins were extracted from transfected cells/treated cells with a buffer containing 0.02 M HEPES (pH 7.9), 0.4 M NaCl, 0.1% NP-40, 10% *v*/*v* glycerol, 1 mM NaF, 1 mM Na_3_VO4, and a protease inhibitor cocktail (Sigma-Aldrich). Cytoplasmic and nuclear proteins were fractionated as previously described [34], and cytosolic/nuclear fractions were evaluated by using the antibody of UCHL3 [36]. Cellular protein extracts were loaded on SDS-PAGE, followed by blotting to PVDF membranes. After blocking in nonfat milk solution, membranes were probed with specific primary antibodies (indicated below) and then incubated with horseradish peroxidase-conjugated secondary antibodies for 1 h. Protein bands were visualized using the ECL chemiluminescence system (Amersham, Buckinghamshire, UK).

Murine adductor muscles were homogenized using the program Protein_1 on a gentleMACS tissue Dissociator (Miltenyi Biotec) [37]. Briefly, tissues were lysated in a buffer containing 50 mM Tris-HCl (pH 7.4), 150 mM NaCl, 1% NP-40, 1 mM EDTA, 0.25% sodium deoxycholate, 10 mM NaF, 10 *μ*M Na_3_VO4, 1 mM PMSF, and protease inhibitor cocktail (10 g/mg aprotinin, 10 g/ml pepstatin, and 10 g/ml leupeptin). Lysates were centrifuged at 14,000 rpm for 15 min, and protein concentrations were measured using Bio-Rad assay kit (Bio-Rad) and immunoblotting was performed as described [38].

MAFG antibody was from GeneTex (GTX114541); HIF-1*α* antibody was from Novus Biologicals (NB100-105). Antibody of FLAG M2 was from Sigma-Aldrich. Antibodies of Nrf2 (number SC-13032), BMI-1 (number SC-390443), HMOX-1 (number SC-136960), tubulin (number SC-8035), UCHL3 (number SC-100340), and vinculin (number SC-7649) were from Santa Cruz Biotechnology Inc., Santa Cruz, CA, USA.

### 2.5. Reverse Transcription-Quantitative Real-Time PCR (RT-qPCR)

Total RNA was extracted using TRIzol reagent (Thermo Fisher SCIENTIFIC), and cDNA was synthesized from one *µ*g of RNA using random primers and iScript cDNA synthesis kit (Bio-Rad Laboratories, Hercules, CA, USA). mRNA levels were quantified using IQ SYBR green supermix (Bio-Rad Laboratories) on the CFX96 real-time system instrument (Bio-Rad). Specific primers located in different exons of the same gene were designed to detect relative mRNA levels. The housekeeping *β*-2 microglobulin or c-ABL genes were used for internal normalization. All the PCR reactions were performed in triplicate, and PCR products were also visualized on agarose gels after ethidium bromide staining. The oligonucleotide sequences are reported in Supplemental Table S2. Relative fold variations were calculated using the 2^−*ΔΔ*Ct^ method by the formula: 2^−(sampleΔCt − controlΔCt)^, where ΔCt is the difference between the amplification fluorescent thresholds of the gene of interest and the internal reference gene/s used for normalization [39].

To detect the levels of mature miR-128, total RNA was prepared with TRIzol reagent (Thermo Fisher SCIENTIFIC) and miR amounts were evaluated by using the TaqMan miRNA assay kit (Thermo Fisher SCIENTIFIC). For normalization of RNA levels, the amounts of small nucleolar RNA RNU6 (Thermo Fisher SCIENTIFIC) were measured [39].

### 2.6. Animal Studies

All experiments involving animals were conforming to the Guide for the Care and Use of Laboratory Animals published by the US National Institutes of Health (NIH Publication 8th edition, update 2011) and were approved by the animal welfare regulation of the University of Naples Federico II, Italy. Wild-type C57BL/6 male mice (age 8 to 9 weeks) were included in the study and maintained under identical conditions of temperature (21 ± 1°C), humidity (60 ± 5%), and light/dark cycle and had free access to normal mouse chow.

### 2.7. Peripheral Ischemia Procedure

Mice were anesthetized with an intraperitoneal injection of 1 ml/kg (50 mg/kg) of a mixture of 50% tiletamine and 50% zolazepam (50 mg/ml tiletamine and 50 mg/ml di zolazepam, Zoletil 100) plus xylazine 5 mg/kg (Sigma-Aldrich). The adequacy of anaesthesia was confirmed by the absence of reflex response to foot squeeze. Hindlimb ischemia was induced as previously described (PI, *n* = 7) [40]. Briefly, the proximal and distal portions of the left femoral artery were ligated and arteriectomy was performed between these two sites. Mice were laid on a heating pad (37°C) under anaesthesia, and their blood flow was measured by a laser Doppler perfusion imager (Perimed, Periscan, USA) in the ischemic and nonischemic limbs before and 0 and 4 hours after surgery, to confirm vascularization impairment after the procedure. SHAM-operated animals underwent the same procedure without femoral artery ligation (SHAM, *n* = 4). All SHAM-operated controls and all mice subjected to peripheral ischemia (PI) survived.

### 2.8. Statistical Analysis

For the statistical analyses, the Student's *t*-test was used; data were considered significant at a value of *p* < 0.05.

## 3. Results

### 3.1. MAFG Is a Novel Direct Target of miR-128

**Figure 1:**
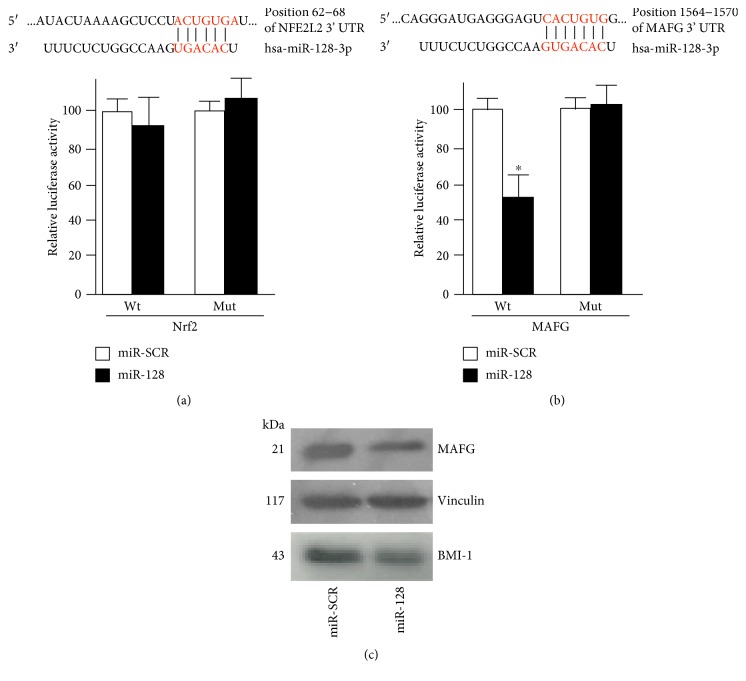
Validation of miR-128 targets by luciferase assay in HEK293 cells. (a) HEK293 cells were transiently transfected with the luciferase construct bearing wild-type (wt) or inverted (mut) 3′UTR fragment holding putative miR-128 target sites of human Nrf2. The alignment of miR-128 with Nrf2-3′UTR is indicated at the top. Cells were simultaneously transfected with either 100 nM of pre-miR-128 (miR-128) or pre-miR negative control (miR-SCR). The pRLSV40 encoding Renilla luciferase plasmid was used as an internal control. Dual luciferase assays were performed as described in the Materials and Methods. Transfections were performed in triplicate, and the ratio (±SD) Firefly/Renilla luciferase activity from three independent experiments were averaged and expressed as percentage of the respective transfections performed with miR-SCR control. ^∗^*p* < 0.05. (b) Luciferase constructs containing wild-type (wt) or mutated (mut) 3′UTR of MAFG with predicted miR-128 site (indicated on the top) were cotransfected with either 100 nM of pre-miR-128 (miR-128) or pre-miR negative control (miR-SCR) in HEK293 cells and luciferase activity quantified as described in (a). ^∗^*p* < 0.05. (c) Western blotting analysis of MAFG and BMI-1 levels on total proteins from HEK293 cells transfected for 24 h with either 100 nM of pre-miR-128 (miR-128) or pre-miR negative control (miR-SCR); vinculin was used as a loading control.

A previous study of Venkataraman et al. reports that miR-128 promotes intracellular ROS increases [31]. Given that Nrf2 signaling is the main driver of the transcriptional program inducing the expression of a wide range of antioxidant genes [3–5], we hypothesized that miR-128 would affect this regulatory pathway. Thus, we used in silico prediction programs to search for putative targets of miR-128 among the components of Nrf2 pathway. TargetScan, miRanda, and RNAhybrid algorithms predicted conserved seed region/s of miR-128 in the 3′UTRs of Nrf2 and MAFG as well as on the Nrf2-regulated gene x-CT (Supplemental Table S1). Since Nrf2 and MAFG are transcription factors and both implicated in the activation of ARE-dependent transcription [8], we performed further analyses on these genes. To assess whether Nrf2 and MAFG are direct targets of miR-128, the entire 3′UTR of Nrf2 and a region encompassing the mir-128 seed sequence of MAFG (250 bp) were cloned into the pGL3-vector downstream of the luciferase gene. Moreover, to validate specificity of miR-128 interaction with 3′UTRs, mutant reporter constructs (mut) harboring the same regions cloned in the opposite orientation were prepared.  Figure 1(a) shows that ectopic expression of synthetic pre-miR-128 slightly decreases the luciferase activity of the construct harboring the 3′UTR region (wt) of Nrf2 in HEK293 cells. In the same conditions, miR-128 overexpression however significantly (*p* < 0.05) reduced the activity of the reporter construct bearing the wild-type 3′UTR (wt) of MAFG compared to miR-SCR-transfected cells (Figure 1(b)). Conversely, mutant MAFG reporter construct (mut) did not respond to miR-128 increases (Figure 1(b)).

To set up a more physiological miR-128 hyperexpression, we also prepared a construct (pCMV-miR-128) that drives miR-128 overexpression from the ARPP21 genomic region (see Materials and Methods). We confirmed that ectopic expression of miR-128 through pCMV-miR-128 (of about 100 folds at 24 h posttransfection, Supplemental Figure S1) in HEK293 cells indeed reduced the luciferase activity of the MAFG-3′UTR reporter construct while there was no effect on the Nrf2-3′UTR construct, very similar to the results obtained with synthetic pre-miR-128 (data not shown).

To validate the above results on the expression of endogenous MAFG protein, we next performed Western blotting analysis on extracts from HEK293 cells transfected for 24 h either with synthetic pre-miR-128 or with pCMV-miR-128.  Figure 1(c) shows that endogenous protein levels of MAFG are significantly reduced in condition of miR-128 overexpression, compared to the negative control-transfected cells. In the same conditions, the downregulation of BMI-1 protein levels, a known miR-128 target, is also detected (Figure 1(c)).

We also observed that miR-128 hyperexpression correlates with MAFG downregulation (Supplemental Figure S2) in physiological/pathological conditions, like differentiation of mouse C2C12 myoblasts [41, 42] or atrophic stimulation of differentiated C2C12 [43]. Taken together, the above reported results suggest that miR-128 represses the expression of the MAFG protein.

### 3.2. miR-128 Influences the Basal Expression of Some MAFG-Regulated Genes

**Figure 2:**
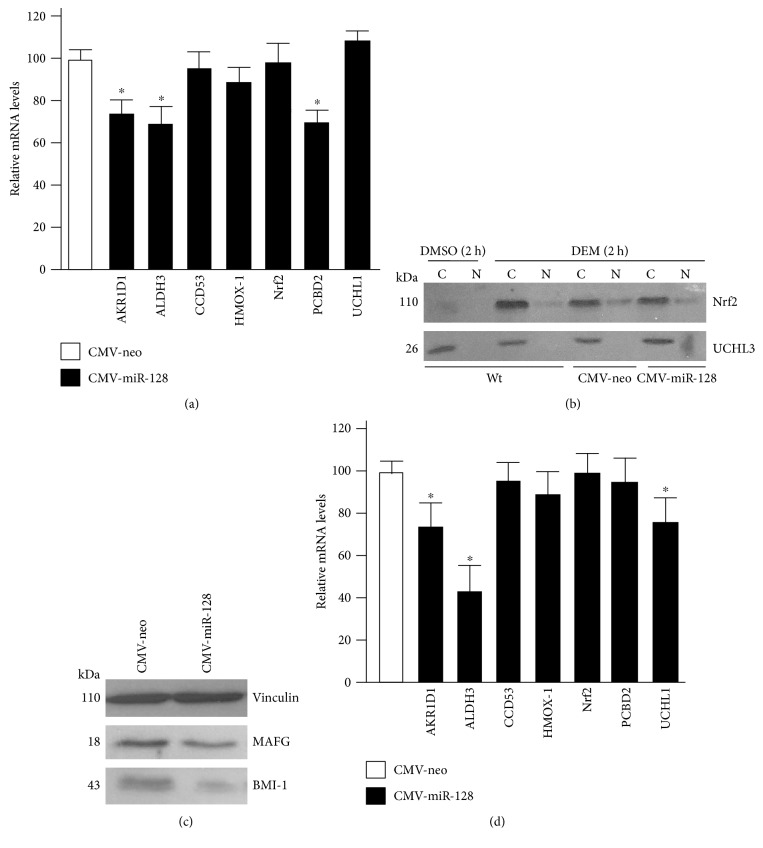
miR-128 downregulates the expression of MAFG and MAFG-regulated genes in human and mouse contexts. (a) HEK293 cells were transfected with either CMV-miR-128 construct or negative control vector (CMV-neo). After 24 h, total RNAs were isolated and the relative mRNA levels of the indicated genes were calculated through quantitative real-time PCR by comparing the levels associated to miR-128 expressing- versus CMV-neo control cells, after normalization with c-ABL. Data were reported as relative to the vector control, which was set equal to 100. Each column in the panel represents the mean ± SD of 3 independent experiments. ^∗^*p* < 0.05. (b) HEK293 cells were transfected with either pre-CMV-miR-128 or negative control (CMV-neo). After 22 h, cells were treated for 2 h with 200 *μ*M DEM. Untransfected HEK293 cells were used as positive control for DEM treatment. Nrf2 protein levels were determined by Western blotting on cytosolic [C] and nuclear [N] extracts. UCHL3 was used as a control of extract preparations. (c) CMV-neo or CMV-miR-128 were transiently transfected into C2C12 cells for 30 h, and Western blotting analysis of MAFG and BMI-1 was performed on total protein extracts; vinculin was used as a loading control. (d) mRNA levels of AKR1D1, ALDH3, CCDC53, HMOX-1, Nrf2, PCBD2, and UCHL1 genes were analyzed as described in (a) at 30 h after transfection in C2C12 cells. Each column in the panel represents the mean ± SD of at least 3 independent experiments. ^∗^*p* < 0.05.

Following the observation that MAFG is negatively regulated by miR-128, we aimed to test the effects of miR-128 on MAFG-dependent transcriptional activity. To this end, we examined the mRNA levels of various MAFG-regulated genes [15, 44] such as AKR1D1, ALDH3, CCDC53, HMOX-1, Nrf2, PCBD2, and UCHL1 upon miR-128 hyperexpression (Figure 2(a)). The results show that in HEK293 cells, the basal expression of AKR1D1, ALDH3, and PCBD2 genes was reduced in conditions of miR-128 overexpression. These results further support a role of miR-128 in the regulation of a set of MAFG-dependent genes.

MAFG is a known basic leucine zipper (bZIP) protein that was shown to interact with other transcription factors, in particular with Nrf2 to activate the expression of numerous genes [7, 15]. Furthermore, MAFG could enhance nuclear retention of Nrf2 [45]. Therefore, we next determined whether the Nrf2 levels are influenced by miR-128 overexpression. Nrf2 is present at very low levels in the cells; thus to stabilize its levels, we exposed wild-type HEK293 and transfected HEK293 cells to low concentration of diethylmaleate (DEM), a glutathione depleting agent [46], for 2 h before harvesting. Western blotting analysis was used to detect the endogenous Nrf2 protein levels after exposure to DEM (Figure 2(b)). In HEK293 control cells, Nrf2 was found substantially increased in the cytosol and also weakly enhanced in the nucleus by DEM exposure. In the same conditions of treatments, cells transfected with miR-128 did not result in reduced accumulation/translocation of Nrf2 compared to cells transfected with empty vector. These results are also in line with the data obtained by 3′UTR luciferase assays that demonstrated that miR-128 is not involved in Nrf2 posttranscriptional regulation.

Since miR-128 has site/s in the 3′UTR of MAFG conserved among mammals (Supplemental Table 1) and since targets of miRNA are cell-type specific, we also examined the effect of miR-128 on the expression of endogenous mouse MAFG using the mouse myogenic C2C12 cell line. The results showed that ectopic expression of miR-128 also promotes downregulation of MAFG protein in the mouse context (Figure 2(c)). In the same conditions, as expected, BMI-1 protein levels were downregulated. In parallel, we evaluated whether the changes observed for MAFG-dependent genes in HEK293 are also present in mouse C2C12 after miR-128 overexpression. As shown in Figure 2(d), the basal mRNA levels of AKR1D1, ALDH3, and UCHL1 genes were reduced in conditions of miR-128 hyperexpression. These results support a role of miR-128 in the regulation of MAFG protein levels and of MAFG-dependent genes also in mouse contexts.

### 3.3. Ectopic Expression of miR-128 Influences the Expression of an Array of ARE-Dependent Genes

**Figure 3:**
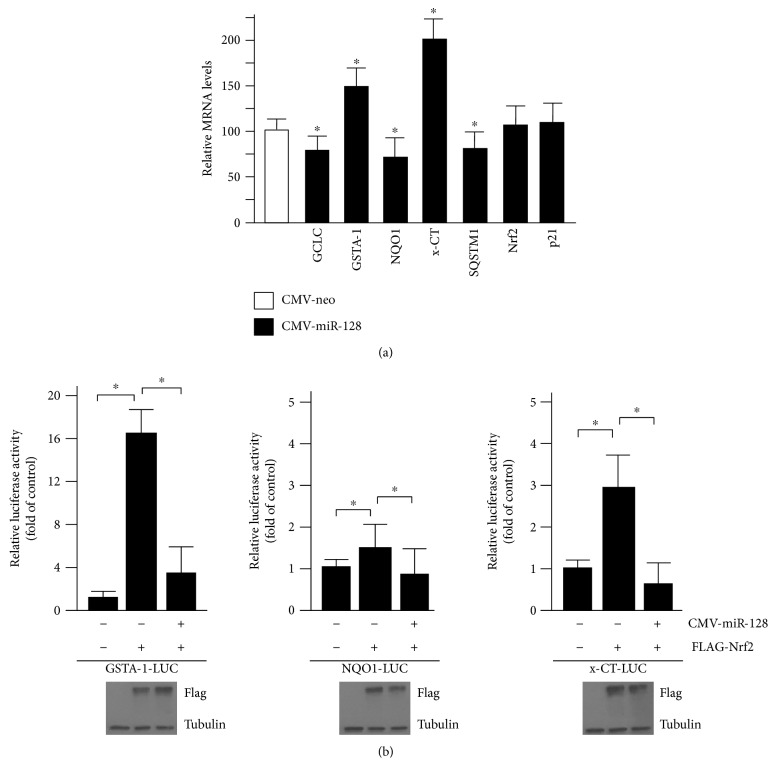
miR-128 provokes modifications of ARE-dependent genes at basal levels and impairs Nrf2 activity. (a) Analysis of GCLC, GSTA-1, NQO1, x-CT, SQSTM1, Nrf2, and p21WAF1 expression in C2C12 cells by RT-qPCR after miR-128 overexpression. mRNA levels in miR-128-transfected cells are expressed as percentage of the levels found in cells transfected with CMV-neo, after normalization with the c-ABL. Each column represents the mean ± SD of 3 independent experiments of transfections. ^∗^*p* < 0.05. (b) The promoter regions of GSTA-1 (GSTA1-LUC) or NQO1 (NQO1-LUC) or x-CT (x-CT-LUC) containing ARE motifs cloned into PGL3 basic vector were transiently cotransfected with pRLSV40 encoding Renilla luciferase, in the presence or absence of miR-128 expressing vector. They were also cotransfected with FLAG-Nrf2 or with equivalent amount of empty plasmid, as indicated. 30 h after transfections, the Firefly/Renilla luciferase activities were assessed and the values were reported as relative to the luciferase activities obtained with the respective LUC construct, which were set equal to 1. The results are representative of three independent experiments, each performed in triplicate. ^∗^*p* < 0.05. Representative Western blotting of FLAG-Nrf2 overexpression is also presented. Tubulin protein levels were used as loading controls.

MAFG as bZIP protein interacts with Nrf2 to facilitate its binding to the ARE consensus within the regulatory regions of numerous cytoprotective genes. To determine whether MAFG downregulation through miR-128 also affects the basal transcription of an array of genes that are typically regulated by the Nrf2:MAFG heterodimer [8, 11], we examined the mRNA levels of representative genes such as GCLC, GSTA-1, NQO1, x-CT, and SQSTM1 in C2C12 cells overexpressing miR-128. The mRNA levels of Nrf2 and p21^WAF1^ were used as controls, since it has been demonstrated that these genes are not dependent on MAFG regulation [8]. As shown in Figure 3(a), the mRNA levels of GCLC, NQO1, and SQSTM1 were significantly (*p* < 0.05) downregulated in C2C12 cells, whereas other Nrf2 target genes such as x-CT and GSTA-1 were upregulated. These findings indicate that protein reduction of MAFG following miR-128 overexpression influences the basal expression of some ARE-dependent genes in C2C12 cells. The above results also suggest that lowered basal expression of GCLC and NQO1 after miR-128 overexpression could explain the ROS increase observed by other authors [31].

Increased levels of Nrf2:MAFG heterodimer activate the transcription of a wide variety of genes containing ARE sequences in their promoters [8]. Therefore, we analyzed the ability of miR-128 to influence the Nrf2 activity on ARE regulatory regions of selected genes. To this aim, we used luciferase reporter constructs, namely, GSTA1-LUC, NQO1-LUC, and x-CT-LUC [34, 35], that in conditions of Nrf2 overexpression strongly induce luciferase activity [34, 35]. Thus, the above LUC constructs were cotransfected in C2C12 cells along with a construct expressing FLAG-Nrf2 or with empty vector, in the absence or presence of miR-128. As shown in Figure 3(b), the luciferase activity driven by the regulatory regions of x-CT and GSTA-1 genes is highly induced after ectopic expression of Nrf2, compared with the control vector, and these effects are completely abolished by the overexpression of miR-128. Even though the Nrf2-mediated activation of NQO1-LUC was less prominent, miR-128 significantly weakened this induction (Figure 3(b)). The expression of ectopic Nrf2 was validated by Western blotting analysis with anti-FLAG antibody.

These results suggested that miR-128 counteracts the Nrf2-mediated induction of ARE-dependent promoters through MAFG downmodulation. The differences observed between the ARE-responsive regions (Figure 3(b)) and the mRNA levels of endogenous GSTA-1 and x-CT (Figure 3(a)) genes could be due to the contributions of additional regulatory regions/transcription factors in the chromatin contexts.

### 3.4. miR-128 Interferes with the DEM-Mediated Induction of ARE-Dependent Genes

**Figure 4:**
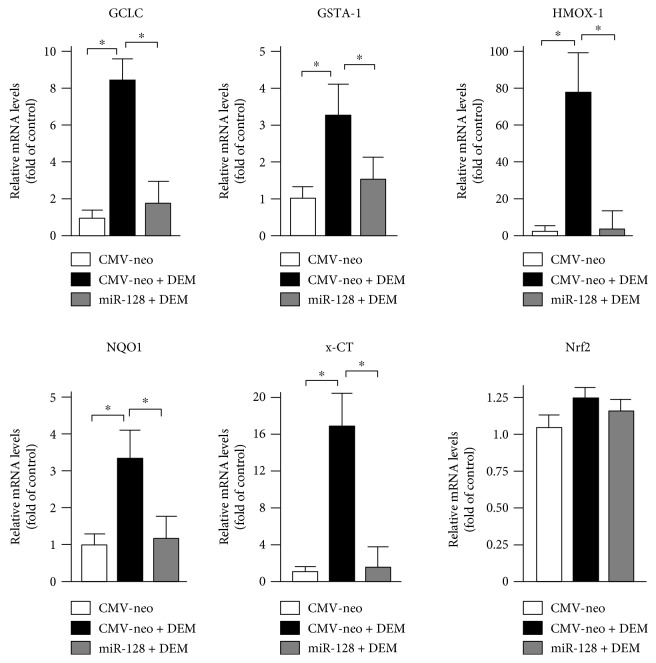
miR-128 interferes with the DEM-mediated induction of ARE-dependent genes. C2C12 cells were transiently transfected with miR-128 expressing construct or with an equivalent amount of empty plasmid. 36 h after transfections, the cells were treated for 3 h with 200 *μ*M DEM and then harvested. The mRNA levels of GCLC, GSTA-1, HMOX-1, NQO1, x-CT, and Nrf2 genes were determined by RT-qPCR on total RNA after normalization with the c-ABL mRNA level. Data are expressed as relative to the values obtained upon transfection with CMV-neo control in the absence of DEM, which were set equal to 1. Each column in the panels represents the mean ± SD of 3 independent experiments. ^∗^*p* < 0.05.

The effects described above led us to investigate whether a reduced expression of endogenous MAFG through miR-128 could result in dysregulation of endogenous stress responses. We selected a group of genes that are responsive to oxidative stress induced by DEM, a well-known inducer of ROS accumulation, by taking advantage of a published list of genes that are responsive to this agent in mouse context [8]. C2C12 cells were transfected with miR-128 or control vectors for 36 h and then exposed to DEM. RT-qPCR analysis was used to determine transcript levels of a small set of ARE-dependent genes, namely, GCLC, GSTA-1, HMOX-1, NQO1, and x-CT. As shown in Figure 4, upon DEM treatments, the endogenous mRNA levels of these genes are strongly induced in C2C12 cells transfected with CMV-neo. In the same conditions of treatments, miR-128 overexpression significantly decreased the DEM-mediated induction of the above genes, when compared to control-transfected cells. In conclusion, miR-128 by downregulating MAFG antagonizes the expression of ARE-dependent genes. The mRNA levels of Nrf2 transcripts, used as control gene, were unaffected [8].

### 3.5. miR-128 Downmodulation Regulates MAFG Increase in Hypoxic Condition Both *In Vitro* and *In Vivo*

**Figure 5:**
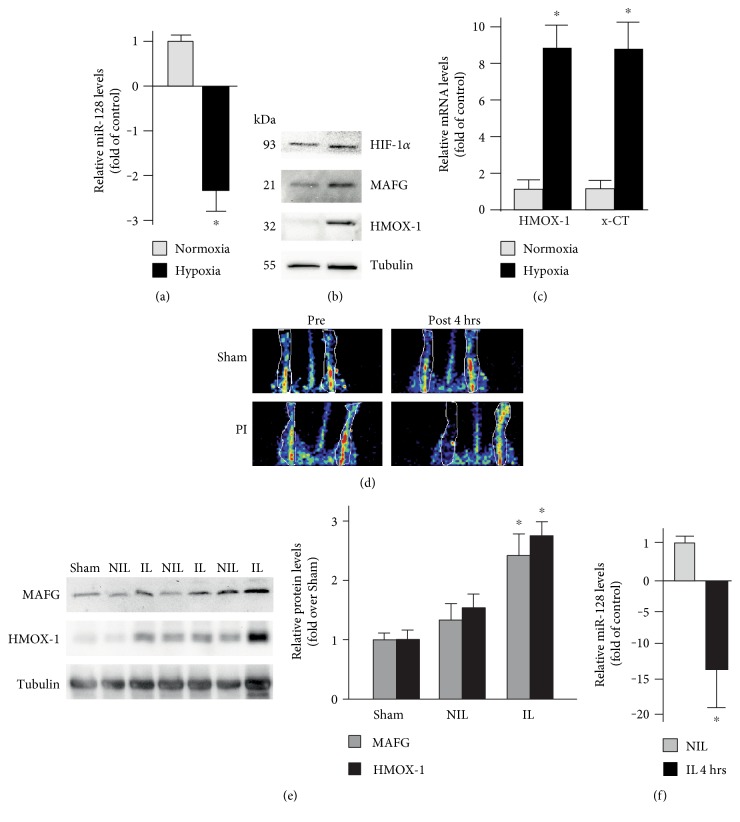
miR-128 downmodulation regulates MAFG increase in hypoxic condition both *in vitro* and *in vivo.* C2C12 cells were exposed to hypoxia (2% O2) for 4 h, and miR-128 levels (a) were determined by RT-qPCR on total RNAs after normalization with the small RNU6. Data are expressed as relative to the values obtained in untreated control which were set equal to 1. Each column in the panels represents the mean ± SD of 3 independent experiments. ^∗^*p* < 0.05. Western blotting analysis (b) of HIF-1*α*, MAFG, and HMOX-1 was performed on total extracts from the same cells, as described in Materials and Methods. Tubulin was used as a loading control. mRNA levels of HMOX-1 and x-CT genes (c) were determined by RT-qPCR on total RNAs, and relative changes were calculated by comparing treated cells versus control, after normalization with c-ABL. Data were reported as relative to the values obtained in normoxia, which were set equal to 1. Each column in the panel represents the mean ± SD of 3 independent experiments. ^∗^*p* < 0.05. Representative laser Doppler analysis (d) of blood flow before and 4 hours after (post 4 h) hindlimb ischemia procedure (*n* = 3-4 mice/group). Representative Western blot analysis and densitometric analysis (e) of MAFG and HMOX-1 protein levels performed on hindlimb muscle lysates from littermates after sham procedure (sham) or not-ischemic limb (NIL) and ischemic limb (IL) 4 h after femoral artieriectomy. Tubulin protein levels were used as loading controls (^∗^*p* < 0.05 versus sham; *n* = 3-4 hindlimb/group). miR-128 levels (f) were determined by RT-qPCR on total RNAs after normalization with the small RNU6. The values obtained in not-ischemic limb (NIL) were set equal to 1. The data are expressed as the mean ± standard error and are representative of 3 independent experiments. ^∗^*p* < 0.05.

Given that MAFG regulates hypoxic responses by interacting with HIF-1*α* [19], we examined the involvement of miR-128-MAFG axis in C2C12 cell responses to hypoxia. First, we estimated the endogenous levels of miR-128 after exposure to hypoxia (2% O_2_) for 4 h. RT-qPCR analysis for mature miR-128 amounts showed significant (*p* < 0.05) decreased levels of miR-128 after hypoxia in C2C12 (Figure 5(a)). To test whether the observed miR-128 changes influence, also MAFG protein levels, we performed Western blotting analysis of MAFG and HMOX-1, which is a common target of HIF-1*α* and Nrf2 transcription factors. The results show that the protein levels of HMOX-1, as expected, are induced following treatment of C2C12 cells within 4 h of low O_2_. Importantly, at the same time, we observed an increase of MAFG protein, which correlates with an enhanced HIF-1*α* expression (Figure 5(b)).

Since hypoxia induces ROS production, we also measured the mRNA levels of selected ARE-regulated genes such as HMOX-1 and x-CT. In this condition, the mRNA levels of these genes are strongly induced after exposure of C2C12 cells to hypoxia (Figure 5(c)). These results indicate that miR-128 downregulation could be important for adjusting the MAFG levels necessary for both HIF-1*α* transactivation and ARE-dependent gene induction in hypoxic conditions *in vitro*.

Finally, we analyzed the role of miR-128-MAFG axis in the ischemic process in vivo. We induced peripheral ischemia (PI) by femoral artery ligation for 4 h in C57BL/6J mice (*n* = 4). Sham-operated animals (*n* = 3) underwent the same surgical procedures without ligation of femoral artery. Hindlimb blood flow was analyzed by ecolordoppler pre- and 4 h after surgical procedures (Figure 5(d)). Western blotting experiments show that MAFG protein levels are significantly induced in the hindlimb muscle lysates (Figure 5(e)). We also investigated whether the upregulated levels of MAFG are inversely correlated to miR-128 amounts. RT-qPCR analysis for mature miR-128 levels show a significant decrease of miR-128 levels (Figure 5(f)).

Overall, these results indicate that miR-128-MAFG axis is an important contributor for responses to hypoxia both *in vitro* and *in vivo*.

## 4. Discussion

miRs play crucial roles in the regulation of gene expression. miRs are considered primarily “fine-tuning” regulators of protein activities, and thus they could balance the antioxidant responses. Recent studies indeed indicate that many miRs called redoximiRs can be either direct or indirect effectors of redox-related pathways [47]. In particular, several evidences demonstrate that, on one hand, Nrf2 itself can be a direct target of miRs and that, on the other hand, Nrf2 modulators, such as Keap1 and Bach1, can be regulated by miRs [5, 48–51]. The transcription factor Nrf2 participates in the adaptive responses to oxidative stress by enhancing transcription of many genes encoding antioxidant/detoxification enzymes, and impaired Nrf2 functions decrease tolerance to oxidative/chemical insults [5].

Herein, we identified a new function of miR-128 as redoximiR, which has a direct role in suppressing MAFG expression. Based on complementary screening for MAFG-dependent genes as well as for ARE-dependent genes functioning through Nrf2:MAFG heterodimer or Nrf2-regulated genes, we demonstrate that miR-128 interferes with the induction of a group of selected genes, namely, GCLC, GSTA-1, HMOX-1, NQO1, and x-CT under oxidative stress. In fact, MAFG acts as positive partner of Nrf2 and its downmodulation by miR-128 consequently affects the Nrf2/ARE pathway.

Deregulated redox signaling and diminished antioxidant defenses represent a major cause of pathophysiological processes including cardiovascular and neurodegenerative diseases, cataracts, diabetes, and cancers, most of which are age related. Therefore, pharmacological/natural strategies addressed to an increase of Nrf2 activity could be beneficial for preventing oxidative stress-related diseases [52–55].

Hypoxia is known to produce ROS, suggesting a cross-talk between HIF-1*α* and the Nrf2 pathways [56]. Since MAFG is related to both of these signalings, we also analyzed the involvement of miR-128 in hypoxic responses. Our results indicate that miR-128-MAFG axis significantly contribute in responses to hypoxia in C2C12 cells and the decrease of miR-128 is parallel with MAFG induction, which consequently could affect MAFG-related genes such as HMOX-1 and x-CT. Of note, the responses observed *in vitro* are also observed during the ischemic process *in vivo*.

Several studies demonstrate the involvement of miR-128 in different cellular processes such as differentiation, apoptosis, senescence, and metabolism [29–32]. Furthermore, miR-128 is found downregulated under hypoxic conditions in human trophoblasts [57] and a recent study implicates a protective role *in vivo* of miR-128 inhibition during myocardial I/R injury [58]. We demonstrate that mir-128 is involved in the modulation of the antioxidant responses *in vitro* as well as in ischemic condition both *in vitro* and *in vivo.* Indeed, repression of miR-128 parallels with MAFG increases that consequently could affect MAFG-related genes such as HMOX-1 and x-CT. Our findings further suggest that downmodulation of miR-128 might contribute to the coordination of the adaptive hypoxic reprogramming that among many others involves genes that are dependent on MAFG, now considered a regulatory hub for numerous transcription factors [8, 15, 19].

Ischemic cardiovascular diseases are a leading cause of morbidity and mortality in developed countries [59]. Even though the detrimental role of ROS generated during ischemia is well demonstrated, an effective antioxidant therapy is still far to be determined. Many studies indeed indicate that Nrf2 protects against cardiovascular diseases albeit reductive conditions also generate ROS (reviewed in [60, 61]). In this context, our work highlights a novel possibility to manage the activities of the Nrf2 signal by miR-128, alone or in combination with other pharmacologic strategies, that in future studies can be considered.

miR-128 has been detected in plasma and its levels are altered in various pathological conditions [62–64], but further studies are required to better clarify the potential role of mir-128 levels as new prognostic and/or diagnostic marker in cardiovascular ischemic diseases.

## Supplementary Material

Supplemental Table S1: List of putative redox-related targets of miR-128 predicted in human by in silico computational analysis. Supplemental Table S2: Sequences of oligonucleotides used for this study. Supplementary Figure S1: Northern blot analysis of miR-128 expression. Supplementary Figure S2: Expression of MAFG under physiological/pathological conditions.

## Abbreviations

ALDH1A1: Aldehyde dehydrogenase 1A1
ARE: Antioxidant-responsive element
AKR1D1: Aldo-keto reductase family 1, member D1
ALDH3A1: Aldehyde dehydrogenase 3 family member A1
CCDC53: Coiled-coil domain containing 53
HMOX-1/HO-1: Heme oxygenase-1
PCBD2: Pterin-4 alpha-carbinolamine dehydratase 2
BACH: BTB and CNC homology
bZIP: Basic-region leucine zipper
CHD8: Chromo-ATPase/helicase DNA-binding protein 8
CNC: Cap ‘n' collar
DNMT3B: DNA methyltransferase 3 beta
EpRE: Electrophile-responsive element
GCLC: Glutamate-cysteine ligase catalytic
GST: Glutathione S-transferase
HIF-1*α*: Hypoxia inducible factor 1 alpha subunit
Keap1: Kelch-like ECH-associated protein 1
miR: MicroRNA
Nrf2/NF-E2: Nuclear factor-erythroid 2
NQO1: NAD(P):Quinone oxidoreductase 1
NRF: NF-E2 p45-related factor
PCR: Polymerase chain reaction
PVDF: Polyvinylidene difluoride
RT-qPCR: Reverse transcription-quantitative real-time PCR
ROS: Reactive oxygen species
sMaf: Small Maf (musculoaponeurotic fibrosarcoma retrovirus transforming gene)
UCHL1: Ubiquitin C-terminal hydrolase L1
UTR: Untranslated region.
