# Supplementary material for: miR-128 Is Implicated in Stress Responses by Targeting MAFG in Skeletal Muscle Cells
Source: Oxid Med Cell Longev. 2017 Sep 12;2017:9308310. doi: 10.1155/2017/9308310 (PMC5613631; doi:10.1155/2017/9308310)
Supplement: Supplementary file 1 — Supplemental Table S1: List of putative redox-related targets of miR-128 predicted in human by in silico computational analysis. Supplemental Table S2: Sequences of oligonucleotides used for this study. Supplementary Figure S1: Northern blot analysis of miR-128 expression. Supplementary Figure S2: Expression of MAFG under physiological/pathological conditions. [file 9308310.f1.doc]

**miR-128 is implicated in stress responses by targeting MAFG in skeletal muscle cells**

Rocco Caggiano, Fabio Cattaneo, Ornella Moltedo, Giovanni Esposito, Cinzia Perrino, Bruno Trimarco, Rosario Ammendola and Raffaella Faraonio

SUPPLEMENTARY MATERIAL

**Supplemental Table S1. List of putative redox-related targets of miR-128 predicted in human by *in silico* computational analysis**

| **Gene symbol** | **Gene name** | **Accession number*** | **Prediction programs (conserved site/s among mammals)** |
| --- | --- | --- | --- |
| ***NFE2L*** | nuclear factor, erythroid 2-like 2 | ENST00000397063.4 | TargetScan, MiRanda  RNAhybrid  (conserved site) |
| ***MAFG*** | musculoaponeurotic fibrosarcoma oncogene homolog G (avian) | ENST00000357736.4HM | TargetScan, MiRanda  RNAhybrid  (conserved site) |
| ***MAFK*** | musculoaponeurotic fibrosarcoma oncogene homolog K (avian) | ENST00000343242.4 | RNAhybrid |
| ***MAFF*** | musculoaponeurotic fibrosarcoma oncogene homolog F(avian) | ENST00000338483.2 | MiRanda, RNAhybrid |
| ***BACH1*** | BTB Domain and CNC Homolog 1 | ENST00000286800.3 | TargetScan, MiRanda  RNAhybrid |
| ***KEAP1*** | Kelch-like ECH-associated protein 1 | ENST00000171111.5 |  |
| ***HMOX1*** | heme oxygenase (decycling) 1 | ENST00000216117.8 | TargetScan, MiRanda, RNAhybrid |
| ***HMOX2*** | heme oxygenase (decycling) 2 | ENST00000570646.1 | RNAhybrid |
| ***GSTA1*** | Glutathione-S-Transferase A1 | ENST00000334575.5 |  |
| ***NQO1*** | NAD(P)H dehydrogenase, quinone 1 | [ENST00000379047.3](http://www.targetscan.org/cgi-bin/targetscan/vert_71/view_gene.cgi?rs=ENST00000379047.3&taxid=9606&members=&showcnc=0&shownc=0&showncf1=&showncf2=&subset=1) |  |
| ***X-CT*** | solute carrier family 7, (cationic amino acid transporter, y+ system) member 11 | ENST00000280612.5 | TargetScan, MiRanda, RNAhybrid  (conserved site) |
| ***GCLM*** | glutamate-cysteine ligase, modifier subunit | ENST00000370238.3 | TargetScan, RNAhybrid |
| ***GCLC*** | glutamate-cysteine ligase, catalitic subunit | ENSG00000001084.6 |  |
| ***SQSTM1*** | sequestosome 1 | ENST00000389805.4 |  |

* accession numbers correspond to those reported in TargetScan 7.1

**Supplemental Tables S1**

**Bioinformatic analysis.** For the identification of miR-128 putative targets, three different algorithms were used, namely TargetScan release 7.1 (http://www.targetscan.org), miRanda (http://www.microrna.org) and RNAhybrid ((http://bibiserv.techfak.uni-bielefeld.de/rnahybrid/welcome.html). The intersection of these programs indicated MAFG and Nrf2 as potential targets for miR-128.

**Supplemental Table S2**: Sequences of oligonucleotides used for this study

HUMAN OLIGOS

| F GSTA-1 | 5’-GGGCTGACATTCACCTGGTG-3’ |
| --- | --- |
| R GSTA-1 | 5’-TTCACTGTGGGCAGGTTACTGA-3’ |
| F HMOX1 | 5’-GGTGACCCGAGACGGCTT-3’ |
| R HMOX1 | 5’-GCGAAGACTGGGCTCTCCT-3’ |
| F NQO1 | 5’-CAGCTCACCGAGAGCCTAGT-3’ |
| R NQO1 | 5’-TAGAGGTCCGACTCCACCAC-3’ |
| F NRF2 | 5’-AACTACTCCCAGGTTGCCCAC-3’ |
| R NRF2 | 5’- GACCGGGAATATCAGGAACAAG-3’ |
| F p21^WAF1^ | 5’-CTGGAGACTCTCAGGGTCGAA-3’ |
| R p21^WAF1^ | 5’-CGGCGTTTGGAGTGGTAGAA-3’ |
| F x-CT | 5’-TGAAATCCCTGAACTTGCGAT-3’ |
| R c-CT | 5’-TCTGGATCCGGGCGCT-3’ |
| F β2-microglobulin | 5’-CCGTGGCCTTAGCTGTGCT-3’ |
| R β2-microglobulin | 5’-TCGGATGGATGAAACCCAGA-3’ |
| F c-ABL | 5′-TGGAGATAACACTCTAAGCATAACTAAAGGT-3′ |
| R c-ABL | 5′-GATGTAGTTGCTTGGGACCCA-3′ |
| F AKR1D1 | 5’-TTGAAGTACCCATGGCCTTT-3’ |
| R AKR1D1 | 5’-TGAAATACGGATGGCACTCA-3’ |
| F ALDH3A1 | 5’-GGAAGAGTCCCTGCTACGTG-3’ |
| R ALDH3A1 | 5’-CCATAGTCCCGGGATTTCTT-3’ |
| F CCDC53 | 5’-CACAAATGGAGCACATCCTG-3’ |
| R CCDC53 | 5’-AGCATCTGGCCTCTCAAGAA-3’ |
| F PCBD2 | 5’-CCTTAAAGCAGCAGGATGGT-3’ |
| R PCBD2 | 5’-TAGGGCAACTCGGGACATAA-3’ |
| F UCHL1 | 5’-CCAGCATGAGAACTTCAGGA-3’ |
| R UCHL1 | 5’-CACAGGAATTCCCAATGGTC-3’ |

Mouse OLIGOS

| F ATROGIN | 5’-CAGCAGCCTGAACTACGACG-3’ |
| --- | --- |
| R ATROGIN | 5’-GGCAGTCGAGAAGTCCAGTC-3’ |
| F MURF1 | 5’-ACCTGCTGGTGGAAAACATC-3’ |
| R MURF1 | 5’-CTTCGTGTTCCTTGCACATC-3’ |
| F GAPDH | 5’-AACATCAAATGGGGTGAGGCC-3’ |
| R GAPDH | 5’-GTTGTCATGGATGACCTTGGC-3’ |
| F GCLC | 5’-TGCGAAAAAAGTGCCCGT-3’ |
| R GLCL | 5’-TGCATTCCAAAACATCTGGAAA-3’ |
| F GSTA-1 | 5′-CAGGTGGCTCCTAGCTGCA-3′ |
| R GSTA-1 | 5′-GGTCTGCGCCAGCTTCA-3′ |
| F HMOX1 | 5’-AGGATTTGTCTGAGGCCTTG-3’ |
| R HMOX1 | 5’-AGGAAGCCATCACCAGCTTA-3’ |
| F MAFG | 5’-TGTGAGTGCCTGCTCACTGT-3’ |
| R MAFG | 5’-GTCAAGCTGGTGCCATTCTC-3’ |
| F NQO1 | 5′-CCCTCAACATCTGGAGCCAT-3′ |
| R NQO1 | 5′-GCGTAGTTGAATGATGTCTTCTCTGA-3′ |
| F NRF2 | 5’-GGCCCAGCATATCCAGACA-3’ |
| R NRF2 | 5’-CCAGGGCAAGCGACTCAT-3’ |
| F p21^WAF1^ | 5’-CCACAGCGATATCCAGACATTC-3’ |
| R p21^WAF1^ | 5’-CGAAGAGACAACGGCACACTT-3’ |
| F SQSTM1 | 5’-AGAATGTGGGGGAGAGTGTG-3’ |
| R SQSTM1 | 5’-TTTCTGGGGTAGTGGGTGTC-3’ |
| F x-CT | 5′-TACCTCAACTTTATTACTGAAGAAGTAGACAA-3′ |
| R x-CT | 5′-TGTCAGTACGTAGCCCACTGTGA-3′ |
| F c-ABL | 5′-GGTATGAAGGGAGGGTGTACCA-3′ |
| R c-ABL | 5′-GTGAACTAACTCAGCCAGAGTGTTGA-3′ |
| F AKR1D1 | 5’-ATGGCGCCTATGTTTACCAC-3’ |
| R AKR1D1 | 5’-CATTGATGGGACATGCTCTG-3’ |
| F ALDH3A1 | 5’-CATCTGACCCCTGTCACCTT-3’ |
| R ALDH3A1 | 5’-CCCATAGTCATGGGACTGCT-3’ |
| F CCDC53 | 5’-AAAAGAACCGTGGCCTTTCT-3’ |
| R CCDC53 | 5’-ATTTGCTGAATCCGGAGAGA-3’ |
| F PCBD2 | 5’-TTCAGATGCACAGTGGTTGA-3’ |
| R PCBD2 | 5’-CATAAAGCCAAACGCCTGAT-3’ |
| F UCHL1 | 5’-GACCATCGGAAACTCCTGTG-3’ |
| R UCHL1 | 5’-GGACAGCTTCTCCGTTTCAG-3’ |

**Northern blot analysis of miR-128 expression.** Total RNA from HEK293 cells transfected for 48 h with pCMV-miR-128 or pCMVneo was isolated using TRIzol Reagent (ThermoFisher SCIENTIFIC) following manufacturer’s instructions. Twenty micrograms of RNA were resolved on 15% polyacrylamide gel with urea 6M and transferred overnight to Hybond N^+^ membrane (GE Healthcare Life Sciences). RNA was fixed by UV exposure (250 nm for 5’). After prehybridization, the membrane was incubated overnight with a probe generated using an oligonucleotide (AAAgAgaCCggTTCACTgTgA**ggACAgAg**) containing the mature anti–miR-128 and an extra-sequence complementary for annealing to the OprobAAA (5’-TTTTTTTTTTCTCTgTCC 3’) that was elongated with Klenow Polymerase (3’-5’exo^-^) (New England BioLabs) using α(^32^P)-dATP (PerkinElmer Inc.) to produce a radioactive adenine tail.

**Expression of MAFG under physiological/pathological conditions.** (A) Mouse C2C12 cells were cultured in DMEM supplemented with 20% FBS. To induce differentiation, medium was replaced to near-confluent cultures (about 80%) with DMEM containing 2% horse serum and cells cultured up to 5 days. Western Blotting analysis of MAFG protein levels was performed on total protein extracts; MyHC (Abcam, USA) was used as control of differentiation and Tubulin as a loading control. Analyses of miR-128 expression levels in control (C2C12 0d) and in 5 days-differentiated C2C12 cells (C2C12 5d) were performed by RT-qPCR as described in the Materials and Methods section of main text. (B) 4 days-differentiated C2C12 cells were treated or not with H_2_O_2_ (100 μM) for 24 h. RT-qPCR experiments for miR-128 levels and Western Blotting analyses for MAFG and BMI-1 protein levels were performed as in (A); Vinculin was used as a loading control. RT-qPCR analyses of ATROGIN1 and MURF1 mRNAs were used as controls of atrophic stimulation in differentiated cells. RT-qPCR assays were performed as (A) and GAPDH was used as internal control. The data are expressed as the mean + standard error and are representative of 3 independent experiments. *p<0.05.
