# Supplementary material for: miR-128 Is Implicated in Stress Responses by Targeting MAFG in Skeletal Muscle Cells
Source: Oxid Med Cell Longev. 2017 Sep 12;2017:9308310. doi: 10.1155/2017/9308310 (PMC5613631; doi:10.1155/2017/9308310)
Supplement: Supplementary file 2 [file 9308310.f2.pdf]

miR-128 is implicated in stress responses by targeting MAFG in skeletal muscle cells

Rocco Caggiano, Fabio Cattaneo, Ornella Moltedo, Giovanni Esposito, Cinzia Perrino, Bruno Trimarco, Rosario Ammendola and Raffaella Faraonio

SUPPLEMENTARY MATERIAL

Supplemental Table S1. List of putative redox-related targets of miR-128 predicted in human by *in silico* computational analysis

Gene symbol	Gene name	Accession number*	Prediction programs (conserved site/s among mammals)
NFE2L	nuclear factor, erythroid 2-like 2	ENST00000397063.4	TargetScan, MiRanda RNAhybrid (conserved site)
MAFG	musculoaponeurotic fibrosarcoma oncogene homolog G (avian)	ENST00000357736.4HM	TargetScan, MiRanda RNAhybrid (conserved site)
MAFK	musculoaponeurotic fibrosarcoma oncogene homolog K (avian)	ENST00000343242.4	RNAhybrid
MAFF	musculoaponeurotic fibrosarcoma oncogene homolog F(avian)	ENST00000338483.2	MiRanda, RNAhybrid
BACH1	BTB Domain and CNC Homolog 1	ENST00000286800.3	TargetScan, MiRanda RNAhybrid
KEAP1	Kelch-like ECH-associated protein 1	ENST00000171111.5	
HMOX1	heme oxygenase (decycling) 1	ENST00000216117.8	TargetScan, MiRanda, RNAhybrid
HMOX2	heme oxygenase (decycling) 2	ENST00000570646.1	RNAhybrid
GSTA1	Glutathione-S-Transferase A1	ENST00000334575.5	
NQO1	NAD(P)H dehydrogenase, quinone 1	ENST00000379047.3	
X-CT	solute carrier family 7, (cationic amino acid transporter, y+ system) member 11	ENST00000280612.5	TargetScan, MiRanda, RNAhybrid (conserved site)
GCLM	glutamate-cysteine ligase, modifier subunit	ENST00000370238.3	TargetScan, RNAhybrid
GCLC	glutamate-cysteine ligase, catalitic subunit	ENSG00000001084.6	
SQSTM1	sequestosome 1	ENST00000389805.4	

* accession numbers correspond to those reported in TargetScan 7.1

Supplemental Tables S1

Bioinformatic analysis. For the identification of miR-128 putative targets, three different algorithms were used, namely TargetScan release 7.1 (http://www.targetscan.org), miRanda (http://www.microrna.org) and RNAhybrid ((http://bibiserv.techfak.uni-bielefeld.de/rnahybrid/welcome.html). The intersection of these programs indicated MAFG and Nrf2 as potential targets for miR-128.

Supplemental Table S2: Sequences of oligonucleotides used for this study

HUMAN OLIGOS

F GSTA-1	5'-GGGCTGACATTCACCTGGTG-3'
R GSTA-1	5'-TTCACTGTGGGCAGGTTACTGA-3'
F HMOX1	5'-GGTGACCCGAGACGGCTT-3'
R HMOX1	5'-GCGAAGACTGGGCTCTCCT-3'
F NQO1	5'-CAGCTCACCGAGAGCCTAGT-3'
R NQO1	5'-TAGAGGTCCGACTCCACCAC-3'
F NRF2	5'-AACTACTCCCAGGTTGCCCAC-3'
R NRF2	5'- GACCGGGAATATCAGGAACAAG-3'
F $p21^{WAF1}$	5'-CTGGAGACTCTCAGGGTCGAA-3'
R $p21^{WAF1}$	5'-CGGCGTTTGGAGTGGTAGAA-3'
F x-CT	5'-TGAAATCCCTGAACTTGCGAT-3'
R c-CT	5'-TCTGGATCCGGGCGCT-3'
F β2-microglobulin	5'-CCGTGGCCTTAGCTGTGCT-3'
R β2-microglobulin	5'-TCGGATGGATGAAACCCAGA-3'
F c-ABL	5′-TGGAGATAACACTCTAAGCATAACTAAAGGT-3′
R c-ABL	5′-GATGTAGTTGCTTGGGACCCA-3′
F AKR1D1	5'-TTGAAGTACCCATGGCCTTT-3'
R AKR1D1	5'-TGAAATACGGATGGCACTCA-3'
F ALDH3A1	5'-GGAAGAGTCCCTGCTACGTG-3'
R ALDH3A1	5'-CCATAGTCCCGGGATTTCTT-3'
F CCDC53	5'-CACAAATGGAGCACATCCTG-3'
R CCDC53	5'-AGCATCTGGCCTCTCAAGAA-3'
F PCBD2	5'-CCTTAAAGCAGCAGGATGGT-3'
R PCBD2	5'-TAGGGCAACTCGGGACATAA-3'
F UCHL1	5'-CCAGCATGAGAACTTCAGGA-3'
R UCHL1	5'-CACAGGAATTCCCAATGGTC-3'

MOUSE OLIGOS

F ATROGIN	5'-CAGCAGCCTGAACTACGACG-3'
R ATROGIN	5'-GGCAGTCGAGAAGTCCAGTC-3'
F MURF1	5'-ACCTGCTGGTGGAAAACATC-3'
R MURF1	5'-CTTCGTGTTCCTTGCACATC-3'
F GAPDH	5'-AACATCAAATGGGGTGAGGCC-3'
R GAPDH	5'-GTTGTCATGGATGACCTTGGC-3'
F GCLC	5'-TGCGAAAAAAGTGCCCGT-3'
R GLCL	5'-TGCATTCCAAAACATCTGGAAA-3'
F GSTA-1	5′-CAGGTGGCTCCTAGCTGCA-3′
R GSTA-1	5′-GGTCTGCGCCAGCTTCA-3′
F HMOX1	5'-AGGATTTGTCTGAGGCCTTG-3'
R HMOX1	5'-AGGAAGCCATCACCAGCTTA-3'
F MAFG	5'-TGTGAGTGCCTGCTCACTGT-3'
R MAFG	5'-GTCAAGCTGGTGCCATTCTC-3'
F NQO1	5′-CCCTCAACATCTGGAGCCAT-3′
R NQO1	5′-GCGTAGTTGAATGATGTCTTCTCTGA-3′
F NRF2	5'-GGCCCAGCATATCCAGACA-3'

R NRF2	5’-CCAGGGCAAGCGACTCAT-3’
F p21^{WAF1}	5’-CCACAGCGATATCCAGACATTC-3’
R p21^{WAF1}	5’-CGAAGAGACAACGGCACACTT-3’
F SQSTM1	5’-AGAATGTGGGGGAGAGTGTG-3’
R SQSTM1	5’-TTTCTGGGGTAGTGGGTGTC-3’
F x-CT	5′-TACCTCAACTTTATTACTGAAGAAGTAGACAA-3′
R x-CT	5′-TGTCAGTACGTAGCCCACTGTGA-3′
F c-ABL	5′-GGTATGAAGGGAGGGTGTACCA-3′
R c-ABL	5′-GTGAACTAACTCAGCCAGAGTGTTGA-3′
F AKR1D1	5’-ATGGCGCCTATGTTTACCAC-3’
R AKR1D1	5’-CATTGATGGGACATGCTCTG-3’
F ALDH3A1	5’-CATCTGACCCCTGTCACCTT-3’
R ALDH3A1	5’-CCCATAGTCATGGGACTGCT-3’
F CCDC53	5’-AAAAGAACCGTGGCCTTTCT-3’
R CCDC53	5’-ATTTGCTGAATCCGGAGAGA-3’
F PCBD2	5’-TTCAGATGCACAGTGGTTGA-3’
R PCBD2	5’-CATAAAGCCAAACGCCTGAT-3’
F UCHL1	5’-GACCATCGGAAACTCCTGTG-3’
R UCHL1	5’-GGACAGCTTCTCCGTTTCAG-3’

Supplementary FIGURE S1

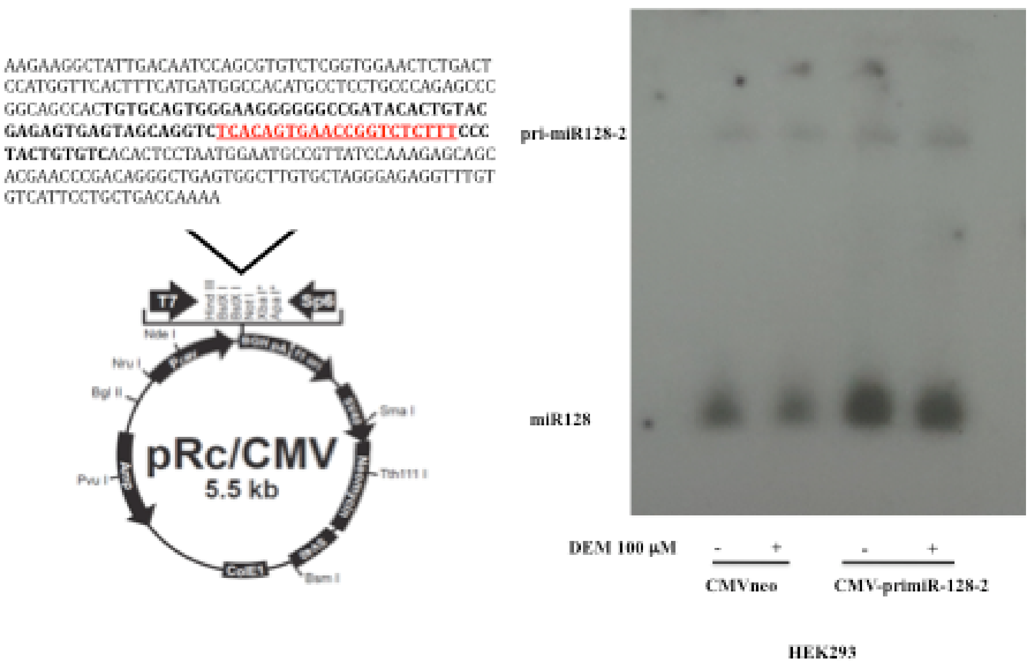

Northern blot analysis of miR-128 expression. Total RNA from HEK293 cells transfected for 48 h with pCMV-miR-128 or pCMVneo was isolated using TRIzol Reagent (ThermoFisher SCIENTIFIC) following manufacturer's instructions. Twenty micrograms of RNA were resolved on 15% polyacrylamide gel with urea 6M and transferred overnight to Hybond N^+ membrane (GE Healthcare Life Sciences). RNA was fixed by UV exposure (250 nm for 5'). After prehybridization, the membrane was incubated overnight with a probe generated using an oligonucleotide (AAAgAgaCCggTTCACTgTgA**ggACAgAg**) containing the mature anti–miR-128 and an extra-sequence complementary for annealing to the OprobAAA (5'-TTTTTTTTTTCTCTgTCC 3') that was elongated with Klenow Polymerase (3'-5'exo$^-$) (New England BioLabs) using α(^{32}P)-dATP (PerkinElmer Inc.) to produce a radioactive adenine tail.

Supplementary FIGURE S2

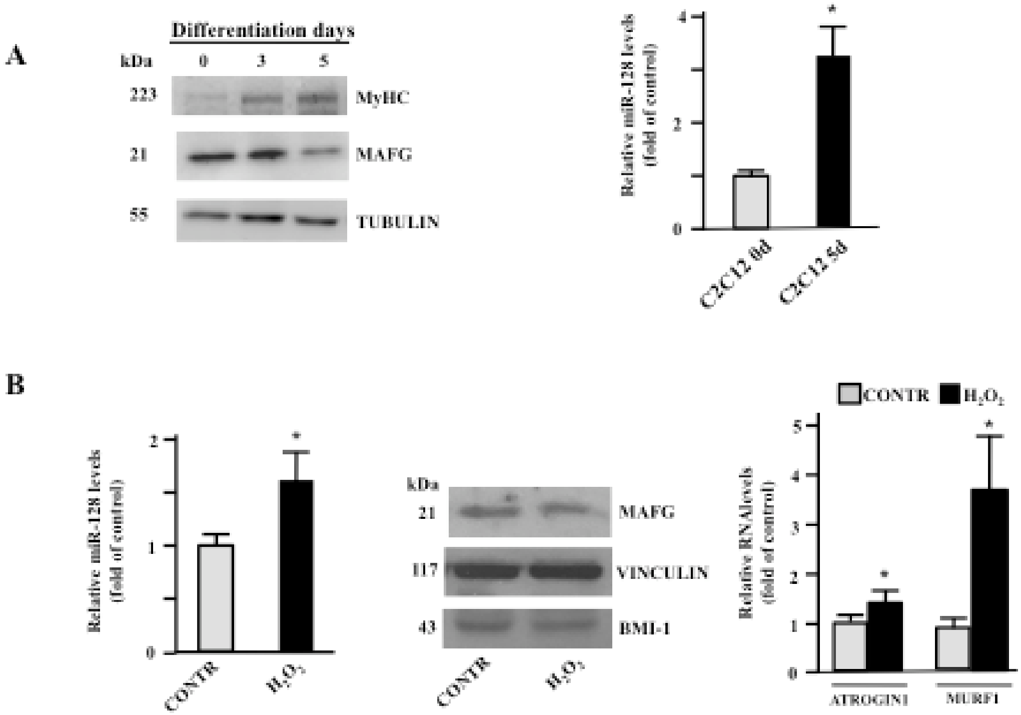

Expression of MAFG under physiological/pathological conditions. (A) Mouse C2C12 cells were cultured in DMEM supplemented with 20% FBS. To induce differentiation, medium was replaced to near-confluent cultures (about 80%) with DMEM containing 2% horse serum and cells cultured up to 5 days. Western Blotting analysis of MAFG protein levels was performed on total protein extracts; MyHC (Abcam, USA) was used as control of differentiation and Tubulin as a loading control. Analyses of miR-128 expression levels in control (C2C12 0d) and in 5 days-differentiated C2C12 cells (C2C12 5d) were performed by RT-qPCR as described in the Materials and Methods section of main text. (B) 4 days-differentiated C2C12 cells were treated or not with H_2O_2 (100 μM) for 24 h. RT-qPCR experiments for miR-128 levels and Western Blotting analyses for MAFG and BMI-1 protein levels were performed as in (A); Vinculin was used as a loading control. RT-qPCR analyses of ATROGIN1 and MURF1 mRNAs were used as controls of atrophic stimulation in differentiated cells. RT-qPCR assays were performed as (A) and GAPDH was used as internal control. The data are expressed as the mean ± standard error and are representative of 3 independent experiments. *$p<0.05$.
